# Spatial dynamics of viral replication drive biphasic lymphoid activation and systemic alphavirus immune responses

**DOI:** 10.1371/journal.ppat.1014509

**Published:** 2026-08-12

**Authors:** Melaina L. Jacoby, Chengqun Sun, Jessica L. Farren, Michelle M. Marti, Alyssa L. Lee, Thomas Donovan, Robyn S. Klein, Dhivyaa Rajasundaram, William B. Klimstra

**Affiliations:** 1 Department of Infectious Diseases and Microbiology, University of Pittsburgh School of Public Health, Pittsburgh, Pennsylvania, United States of America; 2 Department of Immunology and Center for Vaccine Research, University of Pittsburgh, Pittsburgh, Pennsylvania, United States of America; 3 Institute for Quantitative Biomedicine, Rutgers University, New Brunswick, New Jersey, United States of America; 4 Department of Pediatrics, Division of Health Informatics, University of Pittsburgh School of Medicine, Pittsburgh, Pennsylvania, United States of America; 5 Departments of Internal Medicine, Microbiology and Immunology, Western Institute of Neuroscience, Western University, London, Ontario, Canada; National Institute of Allergy and Infectious Diseases Laboratory of Viral Diseases, UNITED STATES OF AMERICA

## Abstract

Systemic type I interferon (IFN-I) and pro-inflammatory cytokine responses are critical for limiting mosquito-borne RNA virus viral replication and disease, yet mechanisms initiating these responses and their linkage to viral tropism for lymphoid tissues are poorly understood. Here, we reveal that virus-cell interactions and specific host signaling pathways in lymphoid tissues are determinants of systemic cytokine induction. Using Venezuelan equine encephalitis virus (VEEV) mutants with defined lymph node (LN) tropism in mice, we show that VEEV LN infection positively associates with systemic IFN-I and other proinflammatory cytokine induction. Maximal responses require coordinated activation of multiple IRF7-dependent Toll-like receptor and RIG-I-like receptor pathways. Combining targeted cell depletion and Visium HD spatial transcriptomics viral gene quantitation, we uncover a spatiotemporal, biphasic innate immune response to VEEV infection, with initial cytokine induction driven by VEEV replication in dendritic cell and monocyte/macrophage subtypes within the LN subcapsular sinus. Surprisingly, active virus replication is curtailed in this LN compartment before 16hpi, when cytokine induction becomes dominated by non-VEEV-replicating bystander cells including plasmacytoid dendritic cells. These findings have direct implications for intervention against viral diseases characterized by lymphoid infection and subsequent spread to sites of terminal disease.

## Author summary

Systemic cytokine responses can determine the outcome of disease after mosquito-borne RNA virus infection, but the mechanisms in peripheral lymphoid tissues that initiate these responses are currently unknown. In this study, we infected mice with Venezuelan equine encephalitis virus (VEEV) to study how severe, acute viral infections replicate in the lymph node, and how that replication is linked to systemic cytokine responses such as type I interferon (IFN-I). We uncovered two waves of cytokine induction in the lymph node early after virus infection. Around 8 hours post infection, actively VEEV-replicating macrophages and dendritic cells in the subcapsular sinus produce IFN-I, which signals to non-VEEV-replicating cells. By 16 hours post infection, plasmacytoid dendritic cells and other non-VEEV-replicating cells produce abundant proinflammatory cytokines and chemokines in response to IFN-I stimulation. Multiple signaling pathways within the cell are necessary for producing this large IFN-I and proinflammatory cytokine response. These findings provide a framework for acute virus infections in the lymph node, identifying IFN-I signaling pathways and early virus replication timepoints as potential targets for antiviral therapeutics or vaccines.

## Introduction

Systemic cytokine responses are commonly induced during RNA virus infection, including Venezuelan equine encephalitis virus (VEEV), a positive-sense arthropod-vectored RNA virus in the *Togaviridae* family that causes symptoms ranging from mild febrile illness to fatal encephalitis [1,2]. After subcutaneous (sc.) inoculation of mice, VEEV exemplifies this systemic cytokine response by spreading to the lymph node draining the infection site (DLN) as early as 4 hours post infection (hpi) [3–5], establishing a highly lymphotropic infection, seeding a high-titer serum viremia [6], and inducing systemic type I interferon (IFN-I) [4,6] and proinflammatory cytokine release [7].

Comparative analysis with eastern equine encephalitis virus (EEEV) supports the connection between lymphoid tissue infection and systemic cytokine production and neuroinvasive disease. EEEV exhibits limited lymphoid tropism, conferred both by efficient heparan sulfate (HS) proteoglycan binding [8] and by host microRNA-mediated virus replication restriction in hematopoietic cells [9–11]. Restricted lymphotropism of EEEV is associated with limited systemic innate immune response induction [11] and unregulated virus replication in the CNS [10], leading to high rates of human mortality [1,2]. In contrast, VEEV induces a robust cytokine response and significant prodromal disease [4,6,7], but encephalitis occurs only in a small fraction of human cases [1]. Thus, the systemic antiviral state elicited by virus interaction with peripheral lymphoid tissue may protect against alphavirus neuroinvasive disease [11,12]. Beyond alphaviruses [4,11,13], lymphotropic viruses trigger stronger systemic cytokine responses, particularly IFN-I responses, compared to their non-lymphotropic counterparts [14–17], suggesting shared mechanisms of immune-mediated pathogenic or protective responses across virus families. However, the cell types involved, timing and mechanisms of protective response induction are poorly understood.

VEEV replicates efficiently in dendritic cells and macrophages *in vitro*, which constitutively express interferon regulatory factor 7 (IRF7), a key controller of virus replication and IFN-I production [4,18–20]. IRF7 deletion ablates systemic IFN-I production after VEEV infection of mice [18,21–23]. However, *in vivo*, the specific pathways for IFN induction versus other cytokines, the ensemble of IFN subtypes contributing to the response, and the contribution of specific cell types and the relationship of virus replication dynamics to quantitative and qualitative aspects of systemic responses are not understood. For example, plasmacytoid dendritic cells (pDCs), which are considered classical “natural IFN-I producers” in response to virus infection, may contribute variably depending on the infection context [24,25], yet it is unknown if these cells are infected by alphaviruses in vivo and whether infected or uninfected pDCs or other myeloid populations contribute to systemic cytokine responses.

To address this knowledge gap, we created mutant VEEV viruses with defined lymphoid tropism by modulating HS-binding efficiency or hematopoietic cell infection. Using the mouse ipsilateral draining popliteal lymph node (PLN) as a general model for virus-lymphoid tissue interactions after a footpad inoculation, we found that the degree of PLN infection is associated with the magnitude of systemic induction of IFN-I and other proinflammatory cytokines, and that this response requires coordinated activation of both toll-like receptor (TLR) and RIG-I-like receptor (RLR) pathways. Finally, we utilized a unique approach to Visium HD spatial transcriptomics by quantitating both virus replication and host response spatially and in specific PLN cells at 8- and 16hpi. These data indicate that the systemic IFN-I and cytokine response is biphasic and initially originates from actively VEEV-replicating myeloid cells of the LN subcapsular sinus, such as type 2 conventional dendritic cells (cDC2s) and macrophage/monocyte lineages, whereas non-VEEV-replicating cells, most prominently pDCs, maintain and expand this cytokine response throughout the remainder of the lymph node after active replication subsides by 16hpi. Furthermore, by associating cell type and infection state with host response, we have identified PLN cells that produce a characteristic cytokine signature of RNA virus infection as well as type III IFN (IFN-III) and IFN-γ antiviral mediators during acute lymphoid tissue infection. These discoveries can lead to (1) early temporal intervention from 8-16hpi and lymphoid-targeted delivery for (2) direct antiviral therapy during peak replication or (3) immune enhancement strategies via IRF7 pathway activation or adjuvants that amplify protective IFN-I responses. Such therapies could be developed against VEEV or EEEV as well as other viruses with initial lymphotropism followed by transit to tissues where disease arises.

## Results

### Efficient VEEV infection of the draining lymph node is associated with high levels of systemic IFN-I induction

To directly associate cell infection and replication efficiency with systemic cytokine induction *in vivo*, we created VEEV mutants with altered replication characteristics in DLNs. We introduced a negatively to positively charged substitution in the E2 attachment glycoprotein of the VEEV ZPC738 strain (E2 E76K) that was previously shown to confer HS binding and attenuate the VEEV TrD strain [26]. Additionally, the EEEV genome contains four miR142-3p binding sites that allow the host microRNA miR142-3p to restrict EEEV replication in hematopoietic cells [11]. To achieve similar restriction of VEEV replication in these cells, we inserted four miR142-3p binding sites or mismatch control sites between the E3 and E2 structural proteins of ZPC738 (miR+ and miR + MM, respectively), similar to previous reporter expression constructions [27]. Standard infectivity and binding assays confirmed that 76K exhibited significantly increased binding to HS, and miR+ replication was significantly restricted by miR142-3p *in vitro* (S1 Fig)*.*

**Fig 1:**
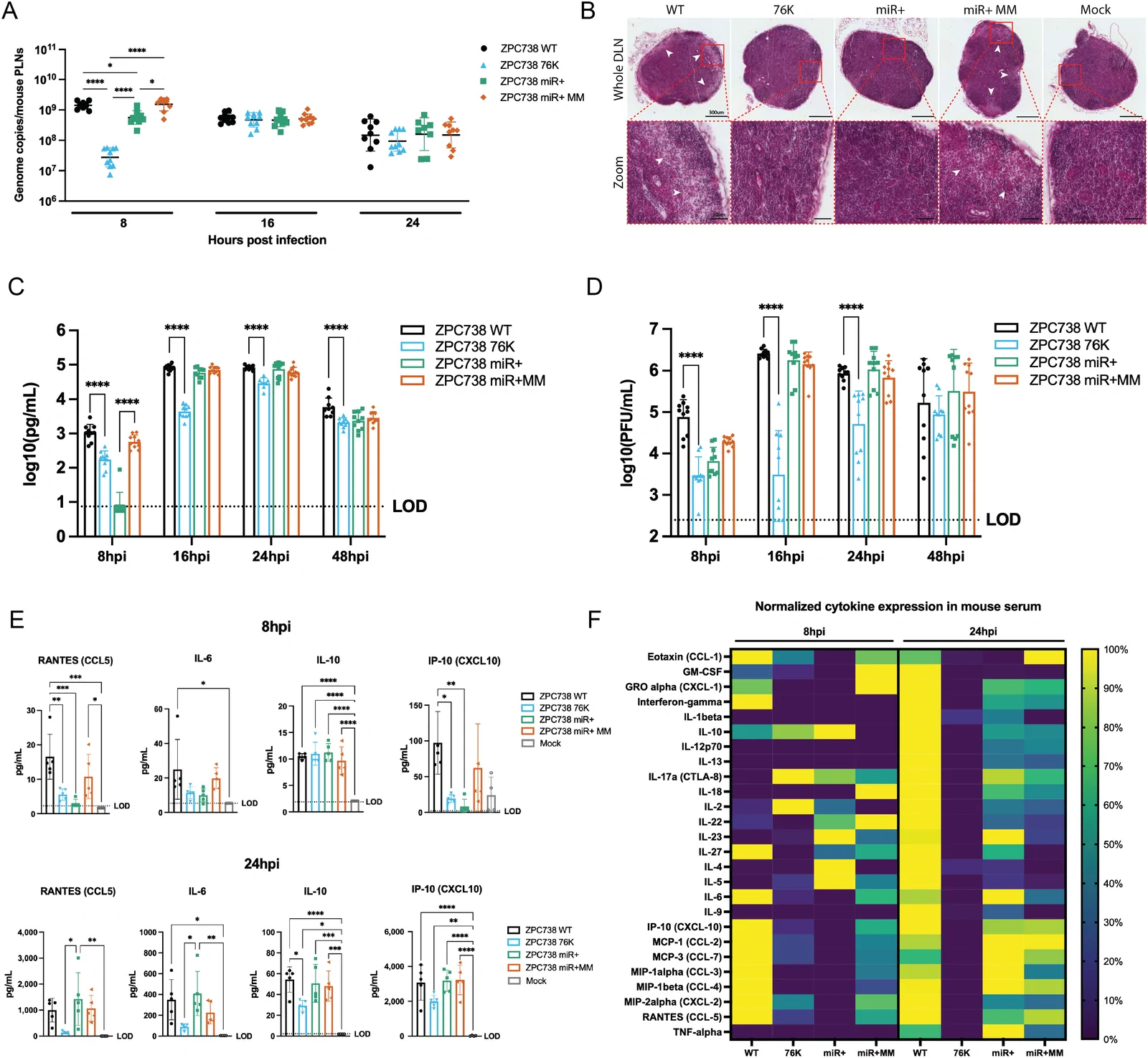
Efficient VEEV infection of the draining lymph node is associated with high levels of systemic IFN-I induction. **(A)** PLNs collected at 8, 16, and 24hpi from infected CD-1 mice, then analyzed for viral load via qRT-PCR (n = 10, 2 independent experiments). **(B)** H&E stains of 8hpi PLNs. Sections of interest are enlarged (bottom), white arrows denote areas of decreased cellularity and apparent cell death (n = 4-6, 1 independent experiment). Scale bar equals 300um (top) and 50um (bottom). **(C-F)** Serum was collected from infected CD-1 mice at 8, 16, 24, and 48hpi. **(C)** IFN-α measured via ELISA and **(D)** infectious viral titers measured via plaque assay (n = 10 mice, 2 independent experiments). **(E-F)** Multiplex ELISA measuring pro-inflammatory cytokines and chemokines in serum at 8 and 24hpi. **(E)** Select cytokines and chemokines or **(F)** relative quantities of all measured cytokines and chemokines. Data normalized to the largest value in each dataset. Mock datapoints included were originally collected and published in Trobaugh et al., 2019 [9]. Each column represents the mean cytokine expression level from 5 individual mice, with minimal inter-individual variation within groups (n = 4-5 mice, 1 independent experiment). Significance determined via 2-way ANOVA **(A, C-D)** or one-way ANOVA within cytokine groups **(E)** with Tukey’s multiple comparison test. Comparisons in **C-D** are between mutants and respective controls (WT/76K; miR + /miR + MM). LOD = limit of detection.

Following footpad inoculation with genomes equivalent to 1000 PFU of the WT virus, both 76K and miR+ mutants showed significantly lower genome copies in the PLNs compared to their respective controls at 8hpi, but not at 16 and 24hpi (Fig 1A). Sequencing confirmed that the mutant viruses did not show reversion or acquisition of compensatory mutations at any timepoint examined. Similar reductions occurred in the spleen through 16hpi (S2A Fig), while footpad levels were minimally affected (S2B Fig), indicating blocked lymphoid transit or replication restriction rather than replication defects at the site of infection. Further, single-round infectious VEEV replicon (VREP) [28] experiments (S2C Fig) and subgenomic-to-genomic ratios (indicating active replication when capsid:nsP2 > 1) in the PLNs and spleen (S2D**-** S2E Fig) further suggest that both the PLN and spleen support initial replication during VEEV infection, but the PLN is the predominant site of replication at 8hpi.

Histologically, both VEEV WT and the miR + MM mutant showed decreased cellularity and density of tissue staining around the subcapsular region of the PLN, indicating apparent cell death, while 76K and miR+ mutants showed minimal pathology similar to the mock (Fig 1B). Since the subcapsular region contains many myeloid cells [29], we conclude that WT and miR + MM replication in miR142-3p-expressing myeloid cells in this region may be causing these cytopathic effects, while miR + is restricted primarily in these cells, and 76K mutant is defective at trafficking to these sites. VEEV protein staining in serial sections was concentrated subcapsularly with punctate staining in other areas (S2F Fig), exhibited quantitatively lower levels in mutant infection (S2G**-**S2H Fig), and colocalized minimally with B cells but not T cells (S2I Fig). Overall, these data confirm that addition of the 76K and miR+ mutations into VEEV confers a restricted replication phenotype in the draining lymphoid tissue.

Next, we sought to determine whether efficiency of lymphoid tissue infection influences the high level of IFN-I and other proinflammatory cytokines or chemokines observed at 8, 16, 24, and 48hpi. Both the 76K and miR+ mutants exhibited significantly lower serum IFNα at 8hpi compared to their respective controls (Fig 1C). While peak production of serum IFNα was observed at 16–24hpi for all viruses, 76K remained significantly reduced compared to the WT throughout infection, whereas miR+ matched miR + MM levels after 8hpi. The 76K mutant also exhibited reduced viremia at 8–24hpi, but miR+ viremia was not significantly different than miR + MM at any timepoint (Fig 1D). This suggests that delayed replication in the lymph node conferred by 76K leads to decreased production of both IFNα and virus titers in serum. Additionally, although systemic virus replication can still occur with miR142-3p-mediated restriction, perhaps through infection of non-myeloid cells, the lack of IFNα at 8hpi suggests that efficient replication in miR142-3p-expressing myeloid cells is necessary for early IFNα production. To further probe the initial source of these systemic cytokine responses, we measured IFNα serum levels 8 hours after infection with VREP at the same genome dose as the parental virus, thus determining how much of the total IFNα response is due to initially infected cells and eliminating FP replication as a factor in DLN seeding. IFNα levels were equal or greater in the VREP-infected mice compared to VEEV virus at 8hpi (S2J Fig), supporting the hypothesis that initially infected cells in lymphoid tissues are largely responsible for the IFNα response detected at 8hpi.

We also investigated other proinflammatory cytokines and chemokines present in the sera from these mice, since these contribute to prodromal disease manifestations after lymphotropic infection [30,31]. Proinflammatory cytokines and chemokines were detectable in the sera at 8hpi, with chemokines like RANTES and IP-10 expressed at higher levels than cytokines like IL-6 and IL-10 (Fig 1E, S1 Table). Both mutants induced lower levels of chemokines compared to their controls at 8hpi, although only 76K was statistically significant (Fig 1E-1F). At 24hpi, all viruses induced robust proinflammatory cytokines and chemokine responses including IFNγ, IL-6, TNFα, and CCL5, but only 76K remained significantly reduced compared to other groups (Fig 1E-1F, S1 Table). This suggests that a delay in replication in the lymph node as conferred by HS binding inhibits high levels of cytokine induction throughout the course of infection, whereas miR142-3p-mediated restriction only provides a transient suppression of cytokine induction biased towards chemokines. This mirrors the results found with IFNα, with which HS binding inhibits IFNα throughout infection, but miR142-3p restriction only impacts induction at 8hpi. Overall, we can conclude that efficient trafficking to and replication within specific cells in draining lymphoid tissues influence systemic IFN-I and other proinflammatory cytokine responses, especially early after infection (~8hpi).

### TLR and RLR pathways cooperatively drive systemic IFN-I and proinflammatory responses

**Fig 2:**
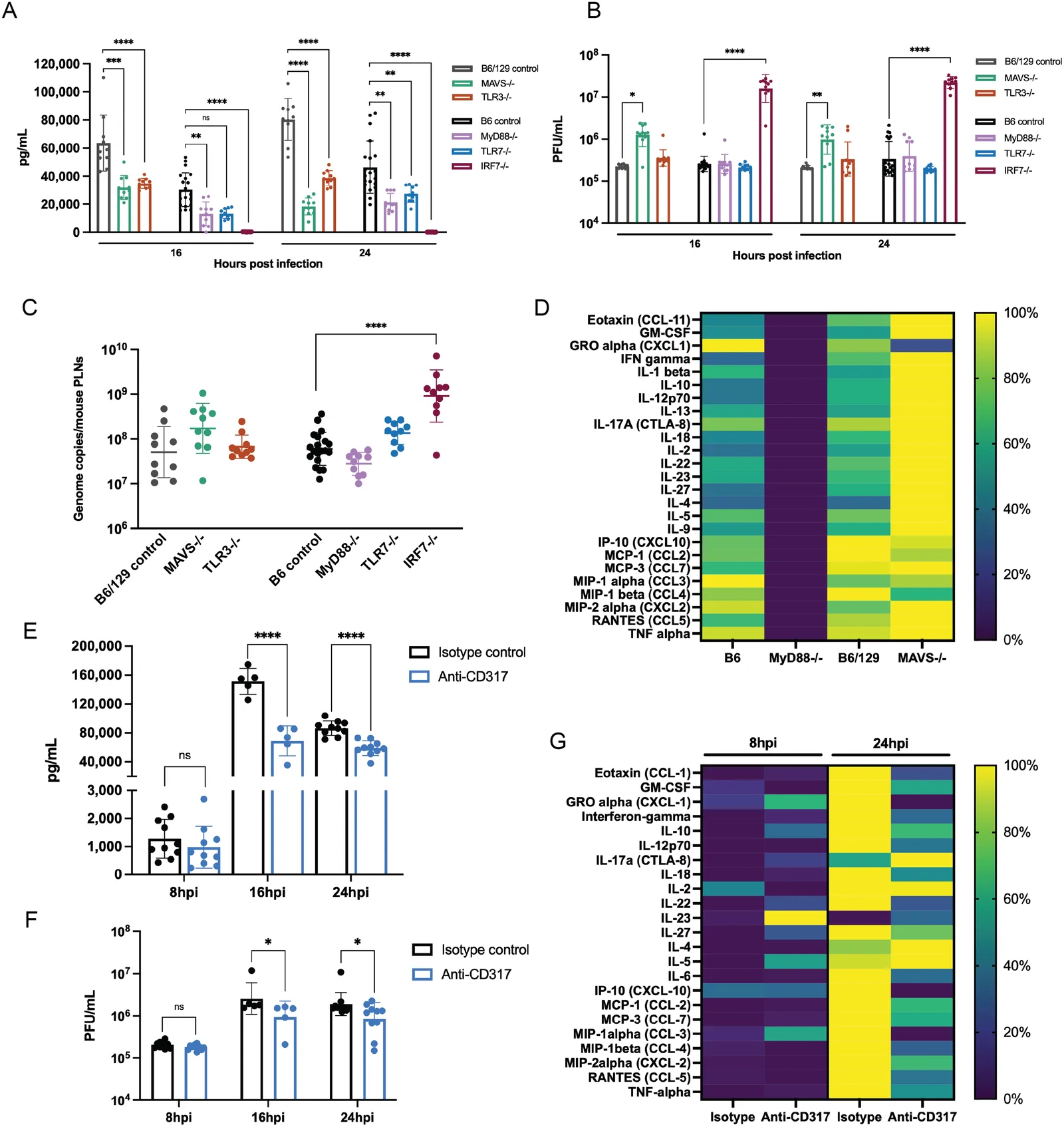
TLR and RLR pathways cooperatively drive systemic IFN-I and proinflammatory responses. **(A-D)** Serum and PLNs were collected from infected knockout and control mice. **(A)** Serum IFN-α measured via ELISA, **(B)** serum infectious viral titers measured via plaque assay at 16/24hpi, and **(C)** viral genome copies measured via qRT-PCR from PLNs collected at 24hpi (n = 10, 2 independent experiments). **(D)** Multiplex ELISA measuring pro-inflammatory cytokines and chemokines in serum at 24hpi (n = 5, 1 independent experiment). **(E-G)** Depletion of CD317 + cells via anti-CD317 antibody. Serum from treated, infected mice collected at indicated timepoints. **(E)** IFN-α measured via ELISA, and **(F)** infectious viral titers measured via plaque assay (n = 10, 2 independent experiments (8, 24hpi); n = 5, 1 independent experiment (16hpi)). **(G)** Multiplex ELISA measuring pro-inflammatory cytokines and chemokines in serum at 8 and 24hpi (n = 5, 1 independent experiment). Data normalized to the largest value in each dataset **(D, G)**. Statistical significance was determined via 2-way ANOVA with Sidak’s multiple comparison test **(A-B, E-F)** or one-way ANOVA with Tukey’s multiple comparison test **(C)**.

VEEV induces extremely high levels of systemic IFNα, as shown in Fig 1. However, detailed insights on which cells and pathways are important to IFN-I responses to virulent VEEV infection have not been previously determined, although previous studies have identified IRF7, not IRF-3, as critical for systemic IFN-I responses [18]. Thus, we performed studies in genetically modified mice to determine whether key adaptor proteins in the TLR or RLR pathways (MyD88 and MAVS) or important pattern recognition receptors (PRRs) in the TLR pathway (TLR3 and TLR7) have an impact on the cytokine responses, viremia, and lymph node replication observed during VEEV infection. We focused on later timepoints (16–24hpi) for these studies, as differences between knockout and WT mice are more biologically informative due to the high levels of IFNα produced during this window. Significant decreases in IFNα induction compared to control mice were observed for all five knockout mouse strains (Fig 2A). In IRF7-/- mice, IFNα was reduced to baseline in accordance with previous studies [32]. IFNα was decreased by over 50% at both timepoints in the MyD88-/- and MAVS-/- mice. TLR3-/- and TLR7-/- mice also exhibited significant decreases compared to controls, albeit to a lesser degree. This indicates that both TLR and RLR pathways contribute to systemic IFNα responses after VEEV infection, and multiple PRRs within these pathways may play additive roles.

The decreases in systemic IFNα induction were not recapitulated in the serum viremia in MyD88-/-, TLR3-/-, or TLR7-/- mice, suggesting that TLR signaling pathways do not influence levels of systemic virus replication (Fig 2B). However, serum viremia exhibited a ~ 5-fold increase in MAVS-/- mice, likely due to lack of cellular control of replication caused by the absence of antiviral effectors normally induced by the RLR pathway. Additionally, IRF7-/- mice exhibited almost 100-fold higher infectious virus in serum at both 16 and 24hpi, indicating that IRF7 signaling is critical for control of virus replication, likely through systemic upregulation of antiviral effector pathways. These trends paralleled quantitation of virus genomes in the lymph nodes, as there was a ~ 2-fold increase in the levels of virus RNA in the PLNs of MAVS-/- mice and a ~ 20-fold increase in IRF7-/- mice, but no differences were observed between TLR3-/-, TLR7-/-, or MyD88-/- mice and their respective controls (Fig 2C). Finally, we measured overall proinflammatory cytokine induction at 24hpi and found that MyD88-/- mice had reduced proinflammatory cytokine and chemokine induction normalized to controls, but MAVS-/- mice experienced a relative increase in cytokines, likely associated with the increased virus replication in these mice (Fig 2D, S2 Table). Therefore, TLR pathways induce both IFNα and systemic cytokine induction, whereas RLR pathways induce IFNα induction and control systemic virus replication.

**Fig 3:**
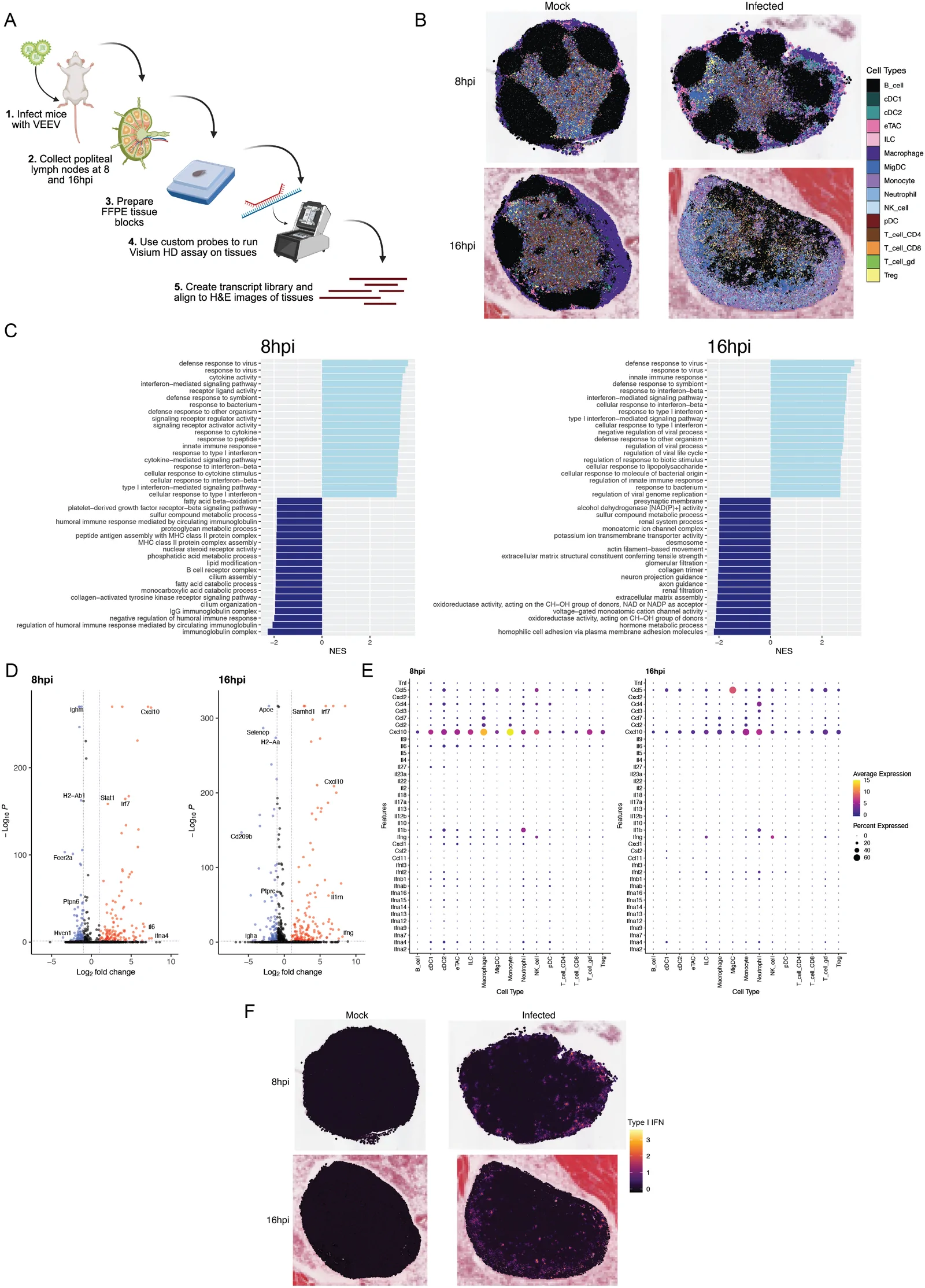
VEEV infection initiates rapid spatial reorganization and proinflammatory responses in the lymph node. **(A)** Schematic of experimental setup. Created in BioRender. Jacoby, **M.** (2026) https://BioRender.com/2w7opfu **(B)** Spatial plots of deconvoluted cell types. **(C)** Bar plot of the top 20 upregulated and downregulated pathways from GO enrichment analysis comparing Infected to Mock at each time point, ranked by Normalized Enrichment Score (NES). **(D)** Volcano plot of differentially expressed genes between Infected and Mock conditions at each time point. **(E)** Dot plot of cytokine expression by cell type. Average gene expression depicted in color, percent of cells expressing that gene depicted as the size of each dot. **(F)** Spatial plot of type I IFN expression. Scale represents log-normalized expression values. Representative lymph nodes used **(B, F)**; or calculated using full dataset (all lymph nodes) **(C-E)**.

**Fig 4:**
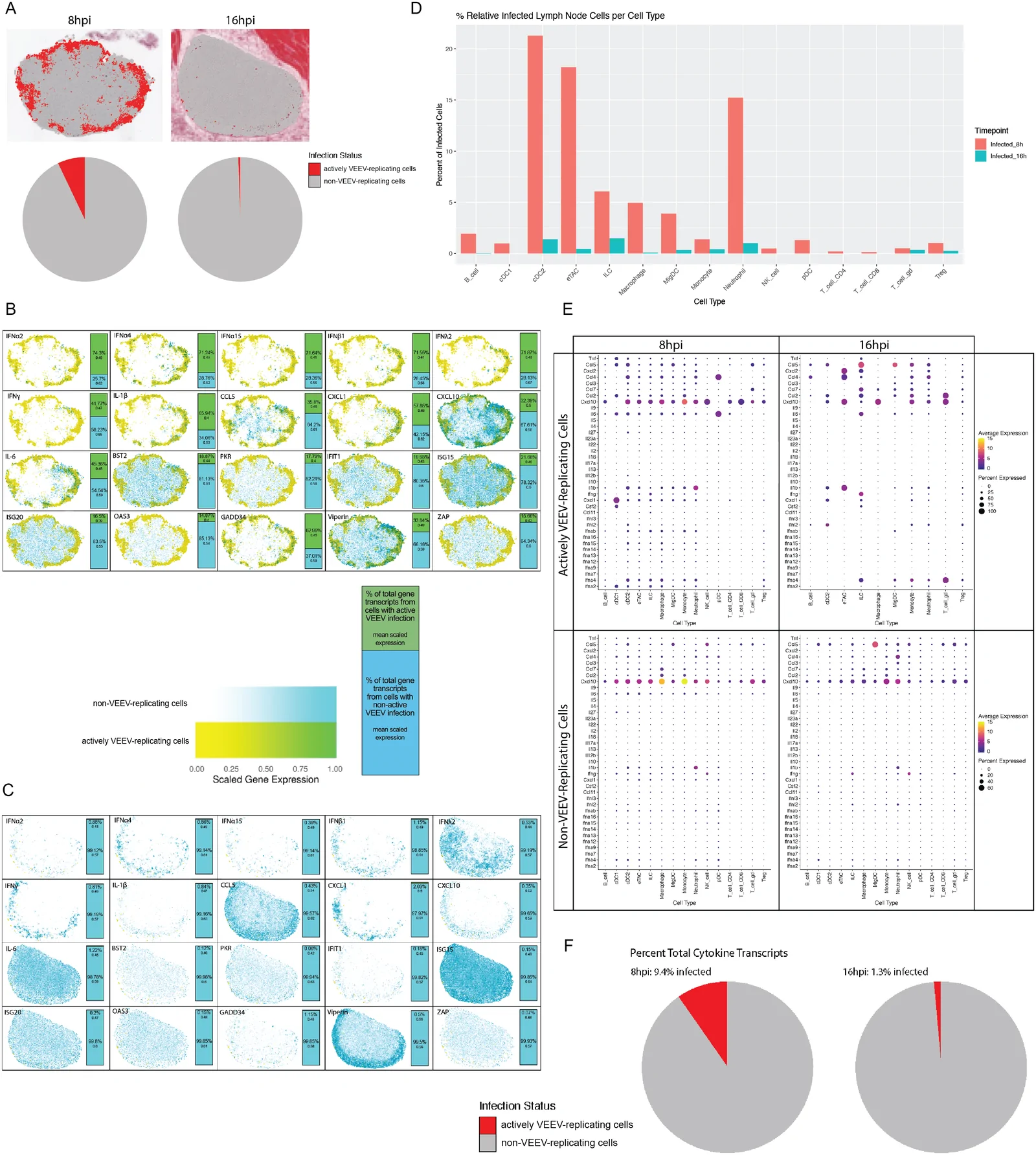
Longitudinal transcriptomics analysis reveals a biphasic cytokine induction profile associated with virus replication dynamics in the DLN. **(A)** Spatial plots showing actively VEEV-replicating cells (red) with E1:nsP4 > 1, UMI(E1) > 50, and UMI(nsP4) > 1. Pie chart represents percent actively VEEV-replicating cells of total. **(B-C)** Expression profiles of select genes (blue) colocalized with infection status (yellow, actively VEEV-replicating; white, non-VEEV-replicating) at **(B)** 8hpi and **(C)** 16hpi. Green indicates that gene expression is coming from actively VEEV-replicating cells. Stacked bar graphs next to each plot indicate percentage of total gene expression contributed to by actively VEEV-replicating (green) versus non-VEEV-replicating (blue) cells. Exact percentages are labeled, as well as mean scaled expression for each group (below). **(D)** Percent relative infection per each cell type in the infected 8hpi and 16hpi samples. **(E)** Dot plot of cytokine expression by cell type and infection status. **(F)** Pie chart showing proportion of VEEV-replicating/non-VEEV-replicating cell contribution to total cytokine induction. Representative lymph nodes used **(A**, spatial plots; **B-C)**; or calculated using full dataset (all lymph nodes) **(A**, pie charts; **D-F)**.

To begin to identify cell types responsible for these IRF7-dependent cytokine responses, we treated mice intraperitoneally (i.p.) with an anti-CD317 antibody to deplete plasmacytoid dendritic cells (pDCs), a cell type prominently implicated in IFN-I responses to viral infections [33,34]*.* We then measured resulting serum IFNα, virus titers, and proinflammatory cytokine responses after VEEV infection. Antibody depletion resulted in ~50% decrease in CD317 + cells in the lymph nodes and spleens at the time of infection (S3A Fig). At 8hpi, we did not observe any differences in serum IFNα, virus titers, or cytokine expression, suggesting that CD317 + cells do not contribute significantly to cytokine induction or virus replication control during initial virus replication (Fig 2E-2G, 3B and S3). However, IFNα and many proinflammatory cytokine levels were decreased by approximately 50% at 16 and 24hpi, indicating a significant contribution of CD317 + cells to cytokine production at these timepoints. Interestingly, virus titers in the depleted mice were significantly lower coincident with the times when lower systemic IFNα was detected (Fig 2E-2F). While pDC infection has not previously been documented for alphaviruses [19], this may suggest CD317 + cells as a target for VEEV infection at later timepoints (Fig 4D) or, alternatively, reflect a generally poor association between modest changes in systemic IFNα levels and viremia as seen in Fig 2A-2B. Overall, these data suggest that robust IFN-I responses to VEEV infection require coordinated activation of multiple signaling pathways and cell types.

### VEEV infection initiates rapid spatial reorganization and proinflammatory responses in the lymph node

To elucidate the cell types in the lymph nodes responsible for IFN-I production and determine contributions of infected versus uninfected cells, we performed 10X Genomics Visium HD spatial transcriptomics on VEEV-infected PLNs at 8 and 16hpi, an interval during which the production of IFN-I and proinflammatory cytokines transitioned from pDC-independent to pDC-dependent. Visium HD technology enables comprehensive spatial transcriptome analysis of mouse tissues at a sub-cellular resolution, providing detailed characterization of cell types involved in virus infection, cytokine induction, and cell-cell signaling while preserving the spatial architecture of the tissue microenvironment. To identify infected cells, we incorporated a set of custom probes for VEEV containing 1–3 viral probe pairs for each viral gene into the mouse transcriptome probes on the Visium HD slides (Fig 3A).

H&E images of infected Visium samples revealed a cleared region of potentially dead and dying cells characterized by decreased cellular density and generally confined to the subcapsular sinus, which was visible at 8hpi and intensified by 16hpi (S4A Fig). Unsupervised spatial clustering, which groups spots with transcriptionally similar profiles, showed altered spatial patterns in infected LNs versus uninfected LNs at 8hpi (S4B Fig), possibly due to cell death caused by viral infection, an influx of inflammatory cells into the lymph node in response to infection, or cell morphology changes resulting from pro-inflammatory cytokine exposure. To identify cell types present in the tissues, we performed reference-based deconvolution using Robust Cell Type Deconvolution [35] with the Immune Dictionary created by Cui et al. [36], successfully delineating 15 different cell types across mock and infected LNs, including B cells, T cells, dendritic cells, macrophages, and other immune cell subsets (Fig 3B). The spatial distribution of these cell types was consistent with canonical lymph node morphology, featuring distinct B and T cell zones, a subcapsular region containing various myeloid cells, and a medullary zone containing abundant macrophage populations [29].

To elucidate broad differences in gene regulation between infected and mock tissue samples, we performed Gene Ontology (GO) enrichment analysis on whole tissues at both timepoints (Fig 3C). As expected, pathways involved in defensive responses to viruses, cytokine activity, responses to interferon, signaling receptor regulator activity, and receptor ligand activity were highly upregulated at both 8hpi and 16hpi. Specifically, upregulated genes such as *Cxcl10, Ifna4, Irf7, Il6,* and *Stat1* demonstrated the proinflammatory skew of the infected tissues (Fig 3D). Conversely, downregulated pathways included general cellular processes such as catabolic processes, ciliary transport systems, GTPase activity, extracellular matrix assembly, and transmembrane transporter activity (Fig 3C). Downregulated genes such as *Fcer2a, Apoe, Selenop,* and *Clu* suggested the suppression of normal cellular function during VEEV infection (Fig 3D), which is in accordance with previous findings that VEEV and other alphaviruses interfere with host cell macromolecular synthesis [18,32].

We also measured the expression of 39 individual cytokine and chemokine transcripts in each identified cell type (Fig 3E), including all IFN subtype genes in the mouse transcriptome probe set, and cytokine and chemokine genes studied in previous protein-level assays (Figs 1F, 2D and 2G). Myeloid cells were the main producers of these transcripts, especially interferon transcripts such as *Ifna2, Ifna4, and Ifna15*, while contributions by B and T cells were minimal. Spatial analysis of these transcripts confirmed a myeloid cell origin, as IFN-I transcripts were located mainly in the subcapsular sinus (Fig 3F). Other proinflammatory cytokine and chemokine transcripts were more widely distributed throughout the lymph node (S4C Fig). Our analysis also revealed an upregulation of type III IFN (IFN-III; *Ifnl3 and Ifnl2*) transcripts in response to infection, localized to similar cell subsets and locations as IFN-I (Figs 3E and S4D). In combination with observed morphological changes, these changes in transcriptional activity underscore our finding that VEEV infection and cell response in the lymph node is rapid and causes robust upregulation of proinflammatory responses in myeloid cells.

### Longitudinal transcriptomics analysis reveals a biphasic cytokine induction profile associated with virus replication dynamics in the DLN

Finally, we aimed to define the transcriptome-based identities of infected cells in the lymph node, then compare their transcriptional profiles to uninfected cells to determine the relative contribution of each group to overall cytokine production. Custom VEEV probes appeared to bind accurately to viral genomes in the tissues, expressing both the expected relative quantity and location of each gene (S5A Fig). Although subgenomic:genomic values have been previously used to indicate active alphavirus replication [37–40] and increased E1:nsP4 values correlated with fewer infected cell numbers as expected (S5B Fig), many infected cells were located in nonpermissive T cell and B cell regions even at high E1:nsP4 (S5C Fig), indicating that subgenomic:genomic measurements alone may be insufficient to accurately predict actively VEEV-replicating cell identities. Thus, multiple parameters were used to determine cellular infection status, including E1:nsP4 values and unique molecular identifier (UMI) thresholds, which measure relative transcript quantity (S5D Fig). To ensure that infection-calling parameters (E1:nsP4 ratio, UMI(E1), and UMI(nsP4)) were accurate, we iteratively evaluated multiple threshold combinations based on empirical ground-truth benchmarks from orthogonal assays. Specifically, virus-specific flow cytometric analysis (FCA) at 8hpi showed that B cells exhibited very low levels of VEEV infection (~2%), and T cells were essentially non-infected (<1%) (S5E**-**S5G Fig). Additionally, immunohistochemical staining (IHC) of VEEV-infected PLNs indicated that ~10% of total PLN area was VEEV+ at 8hpi (S5H Fig). Thus, we selected E1:nsP4 > 1, UMI(E1) ≥ 50, and UMI(nsP4) ≥ 1 as the infection parameters, as they were the least restrictive parameters that recapitulated these results in the Visium HD dataset. We thus define cells above and below this threshold as “actively VEEV-replicating” and “non-VEEV-replicating”, respectively, since some cells below the threshold may have low levels of residual or active infection and cannot be definitively termed “uninfected”.

Spatial plots using these parameters showed that actively VEEV-replicating cells are located almost exclusively in the subcapsular sinus, with approximately 10% of total cells identified as actively VEEV-replicating at 8hpi (Fig 4A). Surprisingly, the number of actively VEEV-replicating cells in this region was reduced to ~1% by 16hpi, suggesting either clearing of virus infection or widespread death of infected cells. These data are reinforced by quantification of viral genome and genome:subgenome ratios in the PLN over time via qRT-PCR, as genome levels and capsid:nsP2 ratios both significantly decreased after 8hpi (Figs 6A and S2D). Additionally, the highest expression of full-length VEEV genomes, measured with nsP4 as a proxy, were localized in the outermost subcapsular region of the LN (S6B Fig), indicating that this region supports the highest levels of replication. These findings emphasize the rapid nature of VEEV interaction with lymphoid tissues and critical contribution of early replication to systemic cytokine responses.

We then examined the spatial relationship of virus infection versus interferon-stimulated gene (ISG) transcripts known to be upregulated during alphavirus infection [41] and the highest-expressed IFN, cytokine, and chemokine transcripts between 8 and 16hpi (Fig 3E). At 8hpi, transcripts such as *Il1b, Cxcl1, Ppp1r15a* (GADD34), and all IFN-I and IFN-III subsets including *Ifna2, Ifna4, Ifna15, Ifnb1,* and *Ifnl2* were highly colocalized with actively VEEV-replicating cells (Fig 4B), which would be expected if virus replication initiated signaling through pattern recognition receptor activation. For these genes, over 50% of the total transcripts were produced by actively VEEV-replicating cells, ranging from 57.85% (*Cxcl1*) to 74.3% (*Ifna2*), representing a positive spatial association with infection. Notably, once activated by VEEV infection, proinflammatory gene expression is not influenced by infection level within an individual cell (S6C Fig). In contrast, other ISG, cytokine, and chemokine transcripts exhibited much lower associations with actively VEEV-replicating cells (14.87-45.36%; *Oas3*, *Il6*, respectively). This likely reflects cytokine signaling through cognate receptors on infected and uninfected cells, which, with ISGs, could be diminished by VEEV blockade of signaling from the IFN-I receptor complex [42]. At 16hpi, very few transcripts (0–2%) were seen to colocalize with actively VEEV-replicating cells, possibly reflecting the dramatic decrease in active replication at this timepoint **(**Fig 4C**)**. For instance, although IFN-I/-III*, Il1b, Cxcl1, and Cxcl10* transcripts were concentrated in the subcapsular region at this time, non-VEEV-replicating cells contributed an overwhelming (≥98%) majority of their total expression. These data suggest that actively VEEV-replicating cells play a major role in IFN and proinflammatory cytokine responses at 8hpi, whereas non-VEEV-replicating cells almost entirely account for these responses at 16hpi.

Next, we determined the percentage of infected cells of each cell type (Fig 4D). At 8hpi, the highest percentage of actively VEEV-replicating cells was observed in conventional dendritic cells, type 2 (cDC2s), extrathymic Aire-expressing cells (eTACs) [43], and neutrophil subsets, while moderate percentages of actively VEEV-replicating cells were observed in innate lymphoid cells (ILCs), macrophages, and migratory dendritic cells (MigDCs; excludes Langerhans cells). A negligible fraction of all other cell subsets was actively VEEV-replicating. Although previous literature exploring lymph node cell tropism during VEEV infection shows similar findings, no evidence has been found for neutrophil infection by VEEV [4,19,44,45]. In concordance with our data, FCA of lymph nodes infected with eGFP-expressing VEEV identified CD11c+ cells, CD11b+ cells, and CD11b^+^F4/80^-^Ly6C^+^ inflammatory monocytes/neutrophils at 8hpi as eGFP+ (S5E Fig). This indicates that neutrophils may indeed be infected *in vivo*, but it is important to note that both transcriptomic and flow cytometric results may instead represent phagocytosis of cell debris by neutrophils, including active replication complexes, and not genuine infection [46]. Despite this limitation, orthogonal data supports our conclusion that myeloid cells, particularly dendritic cells, are the primary target of VEEV infection in lymph nodes *in vivo*.

**Fig 5:**
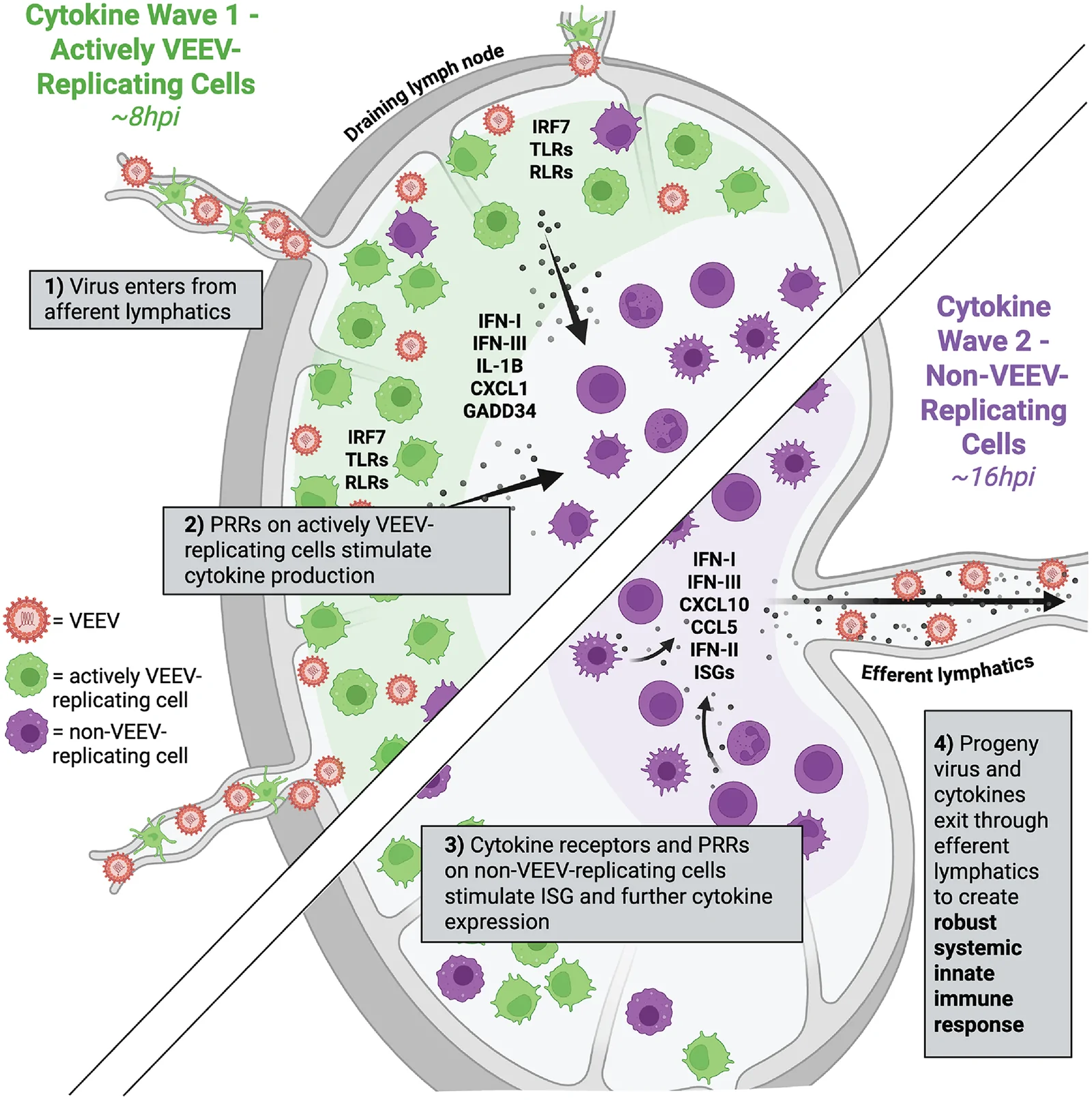
Efficient alphavirus infection in the lymphoid tissues initiates a biphasic innate immune response. Schematic depicting high lymphoid infection with VEEV (top) contrasted with low lymphoid infection (bottom). Actively VEEV-replicating myeloid cells in the subcapsular sinus utilize pattern recognition receptor pathways to recognize viral RNA and produce proinflammatory cytokines and chemokines in the first response wave (~8hpi). Non-VEEV-replicating bystander cells sustain and amplify the cytokine response during the second response wave (~16hpi), releasing these cytokines alongside progeny virus via the efferent lymphatics, leading to systemic infection and proinflammatory innate immune responses. VEEV = red virus particles; actively VEEV-replicating cells = green cells; non-VEEV-replicating cells = purple cells; IRF7 = interferon regulatory factor 7; TLR = toll-like receptor; RLR = RIG-I-like receptor; ISGs = interferon-stimulated genes. Created in BioRender. Jacoby, **M.** (2026) https://BioRender.com/s26lx8f.

We also examined the expression of proinflammatory cytokines and chemokine transcripts in both actively VEEV-replicating and non-VEEV-replicating cells at each timepoint (Fig 4E-4F), using the full systemic innate immune response panel identified in Fig 3E. At 8hpi, proinflammatory chemokine transcripts such as *Cxcl10*, *Ccl4,* and *Ccl5* were expressed by virtually all cell types, whereas proinflammatory cytokine transcripts such as *Il6* and *Il1b* were upregulated particularly by myeloid cells of both VEEV-replicating and non-VEEV-replicating groups (Fig 4E). These data support previous studies identifying many of these genes, especially proinflammatory chemokines, as upregulated on both the transcriptomic and protein levels during VEEV infection [7,47–49]. However, a stark difference was observed between VEEV-replicating and non-VEEV-replicating cells for IFN-I, wherein a higher percent of actively VEEV-replicating cells upregulated IFN-I genes at 8hpi compared to non-VEEV-replicating myeloid cells. At 16hpi, a high percentage of actively VEEV-replicating cells still expressed many of these transcripts; however, the small population size of these cells at 16hpi implies that most transcripts are present in non-VEEV-replicating cells (Fig 4E). Thus, cytokine transcript expression was dominated by non-VEEV-replicating cells, which expressed upregulated proinflammatory chemokine and cytokine transcriptomic profiles. Indeed, quantification of VEEV-replicating/non-VEEV-replicating cell contributions to total cytokine transcript numbers (Fig 4F) using the full systemic panel (Figs 3E and 4E)*,* indicates that 9.4% of total cytokine and chemokine transcripts are contributed by actively VEEV-replicating cells at 8hpi, compared to 1.3% of transcripts at 16hpi. Although all IFN-I and IFN-III subsets are primarily expressed by actively VEEV-replicating cells at 8hpi (Fig 4B), these aggregate data (Fig 4F) are likely skewed by high expression of chemokines such as *Cxcl10* and *Ccl5* in both VEEV-replicating and non-VEEV-replicating cells. Overall, these data support the conclusion that a two-wave cytokine response to VEEV infection is elicited from infected lymphoid tissue, first directed by actively VEEV-replicating myeloid cells and, subsequently, dominated by non-VEEV-replicating myeloid cells including but not limited to pDCs, neutrophils, monocytes, and NK cells (Fig 5).

## Discussion

The protective role of IFN-I in neurotropic viral infections has broad implications for understanding disease pathogenesis and developing therapeutic interventions. Systemic IFN-I is believed to mitigate viral replication and severe injury in the CNS [11,13–17,49–52] through activation of NF-kB[50], inhibition of replication-induced neuronal death [12,14,15] or restriction of neuroinvasion via the blood brain barrier [51,52]. Consistent with our observation that infected cells in the subcapsular sinus of the DLN spearhead cytokine responses to VEEV infection, studies with West Nile Virus (WNV) have utilized non-propagative virus-like particles *in vivo* to suggest that initially infected cells in lymphoid tissues rapidly produce robust systemic IFN-I responses [53]. Similar mechanisms may occur during vesicular stomatitis virus (VSV) infection, where efficient replication in CD169 + macrophages promotes IFN-I production by these cells and by pDCs in the lymphoid tissues [16,17]. However, the above studies did not spatially associate virus infection in specific cells with host gene upregulation. Our spatial transcriptomic analyses implicate actively VEEV-replicating myeloid cells, including CD169 + macrophages, as contributors to early IFN-I induction by actively VEEV-replicating cells during VEEV infection. For the first time, we co-localize and quantitate virus replication and IFN production in specific cells and structures within the LN. Furthermore, we observed IFN-III gene induction in LN cells suggesting that multiple IFN subtypes contribute to the acute systemic response. These data suggest a conserved, protective mechanism of critical innate immune activation in lymphoid tissues. Thus, therapeutics targeting lymphoid tissue infection during the critical early window, including adjuvants or immune modulators delivered directly to lymphoid tissues, could amplify the protective IFN-I response before CNS invasion occurs.

The coordination of innate immune response induction pathways during antiviral responses varies dramatically across viral families, necessitating pathogen-specific therapeutic strategies. For example, TLR pathways are important for control of chikungunya virus (CHIKV), as loss of MyD88 signaling increases viremia [54,55]. Conversely, MyD88-dependent pathways stimulate IFN-I during herpes simplex virus type I (HSV-1) and protect against severe Ross River virus (RRV) infection, but do not impact viral loads in either case [56,57]. RLR pathways exhibit similar virus-specific heterogeneity. Although RLR pathways trigger innate immune responses during infections with almost all major virus families [58], MAVS-dependent pathways control viremia in CHIKV infection [55,59]; whereas, hepatitis C virus (HCV) cleaves MAVS during early infection, rendering it unable to control viremia [60,61]. In the current study, we found that VEEV relies on a previously unrecognized, IRF7-regulated balance of both TLR and RLR pathways to induce systemic IFN-I, diverging from models derived from arthritogenic alphaviruses. TLR pathways preferentially drive broader proinflammatory cytokine and chemokine responses and may be ideal targets for immunomodulatory interventions, while RLR pathways restrict systemic VEEV replication and could be targets for antiviral therapies. Overall, these data reinforce that an understanding of signaling pathway contributions is essential for developing virus-specific therapeutics, rather than assuming universal mechanisms across related pathogens.

Finally, signaling interactions between infected and uninfected cells in the lymph node during virus infection have been previously postulated but never confirmed. *In vitro*, myeloid cells such as cDCs and macrophages are the primary targets of VEEV replication [4] and producers of IFN-I[32], but it has remained unclear whether these cells also initiate IFN-I responses *in vivo* or signal to bystander cells. A previous study used an mRNP tagging system and non-propagative VEEV vectors to assess innate responses in the DLN*,* associating early host gene activation with infected cells [62]. Although informative, this study captured a limited subset of genes and utilized single-round infectious replicon particles, precluding analysis of the contribution of virus replication kinetics and dissemination in the process. Our work provides a comprehensive, *in vivo* demonstration of biphasic cytokine induction using propagation-competent natural VEEV infection and high-resolution spatial transcriptomics co-registered with protein-level analyses.

Analysis of lymphoid tissues is fundamentally difficult due to densely packed cellular architecture, especially in pseudo-single-cell methods such as Visium HD that capture transcripts in grid-like “spots”, not within defined cell boundaries. This can lead to erroneous detection of virus infection in non-permissive cells due to phagocytosis of infected cell debris or misassignment of viral transcripts from adjacent infected cells. We believe that a conservative definition of active VEEV infection, refined by conventional flow cytometric and immunohistochemical techniques, has mitigated these effects. However, this conservative definition may misclassify cells with lower levels of active replication as “non-VEEV-replicating cells”, which we acknowledge as a limitation of our analysis. Additionally, while cell-type deconvolution is a powerful tool for scRNA-seq and spatial transcriptomic datasets, precision decreases for cell subsets that exhibit subtle differences in transcriptomic profiles, potentially leading to mis-annotations, especially in cases like the current study where the reference dataset differs from our treatment and fixation conditions [63]. Such issues may have led to our interpretation that neutrophil subsets are infected by VEEV. This observation will require additional in vivo and in vitro assessment. However, we assert that our central conclusions are not compromised by these limitations, as orthogonal experimental methods in the current studies support the conclusions about the identities and roles of cell types in cytokine signaling mechanisms.

Our spatial analyses prove that VEEV elicits an early wave of IFN-I and proinflammatory cytokine production from actively VEEV-replicating myeloid cells in the subcapsular sinus, followed by a robust second wave produced by non-VEEV-replicating cells throughout the DLN as viral replication wanes. Several mechanisms may underlie signaling to non-VEEV-replicating cells beyond canonical cytokine signal transduction, including recognition of viral RNA by pDCs via TLR7/9 [23], DAMP receptor engagement by infected cell debris [64], or stimulation of heat shock protein receptors [65]. Our findings also place VEEV within a broader motif observed in unrelated virus families. During modified vaccinia virus Ankara (MVA) infection in the LN, infected macrophages and cDCs release proinflammatory chemokines that recruit uninfected pDCs to produce IFN-I around 8hpi [66]. Influenza A virus (IAV) was also found to elicit a two-phase proinflammatory response motif in the lung, with a first wave of proinflammatory factors spearheaded by infected neutrophils, followed by a second wave from uninfected macrophages after IAV replication waned [67]. Our data suggest that this biphasic process may represent a conserved mechanism of the innate immune system in response to acute viral infection. Identification and characterization of this common innate immune process provide pathways for sculpting early virus-immune cell interactions towards protective systemic responses that improve vaccines and therapeutics.

## Materials and methods

### Ethics statement for mouse studies

All mouse experiments were carried out in the Regional Biocontainment Laboratory at the University of Pittsburgh’s Center for Vaccine Research under protocol 24105846 approved by the Institutional Animal Care and Use Committee of the University of Pittsburgh. Studies were performed in accordance with AAALAC-approved institutional guidelines for animal care and use. Female 4–6-week-old mice were used for all studies. Female mice have been the primary sex historically used in alphavirus studies and within our laboratory. [13,26,49,68]. Wild-type mice were purchased commercially and included the following strains: CD-1 (Charles River), C57BL6/J (Jackson Laboratories), and B6129SF1/J (Jackson Laboratories). The following knockout mice were purchased commercially from Jackson Laboratories: B6;129-*Mavs*^*tm1zjc*^/J (MAVS-/-), B6.129P2(SJL)-*Myd88*^*tm1.1Defr*^/J (MyD88-/-), B6;129S1-*Tlr3*^*tm1Flv*^/J (TLR3-/-), and B6.129S1-*Tlr7*^*Tm1Flv*^/J (TLR7-/-). IRF7-/- mice were bred in-house at the University of Pittsburgh Animal Facility. All mice were housed in facilities that maintained a temperature range of 20-26.1°C and a 30–70% relative humidity range on a 12:12 light:dark cycle with water and food provided ad libitum. Mice were inoculated with virus under anesthesia that was induced and maintained with isoflurane, then monitored until study endpoint or approved euthanasia criteria. All efforts were made to minimize animal suffering.

### Cell lines

Baby hamster kidney (BHK-21 [ATCC CCL-10]) cells were maintained in RPMI-1640 medium (Corning) supplemented with 10% (v/v) heat-inactivated donor bovine serum (DBS; Gibco) and 10% (v/v) tryptose phosphate broth (Moltox). Chinese hamster ovary wild-type (CHO-K1 [ATCC CRL-61], mutant GAG-deficient (pgsA-745 [ATCC CRL-2242]), and HS-deficient (pgsD-677 [ATCC CRL-2244]) cells were maintained in Ham’s F-12 medium (Corning) supplemented with 10% (v/v) heat-inactivated fetal bovine serum (FBS; Gibco). RAW 264.7 [ATCC TIB-71] cells were maintained in DMEM medium (Corning) with 10% (v/v) fetal bovine serum (FBS; Gibco). All media contained 2mM L-glutamine, 100 U/mL penicillin, and 0.1 mg/mL streptomycin.

### Viruses and replicons

All viruses and replicons used in this study were generated from cDNA clones. VEEV ZPC738 cDNA clones were generously provided for these studies by Scott Weaver (University of Texas Medical Branch, Galveston, TX). QuikChange II XL mutagenesis kit (Agilent) was used to make E2 76K point mutation in ZPC738 (ZPC738 E2 76K). To make ZPC738 miR+ virus, MiR142BC-pUC57 was synthesized from Genscript. Then the QuikChange II XL mutagenesis kit (Agilent) was used to insert a PCR fragment of mir142BC TaV at the capsid/E3 junction that encoded the first five amino acids of E3 fused in frame to reporter genes followed by the TaV 2A-like protease. To make ZPC738 miR + MM virus, MiR142BC-MM-pUC57 was synthesized from Genscript. Then the QuikChange II XL mutagenesis kit (Agilent) was used to insert a PCR fragment of MiR142BC-MM TaV. For chimeric viruses SINV/ZPC738 WT and E2 76K, A PCR fragment of ZPC738 structural proteins with eGFP TaV was inserted into SINV replicon with Xba I and Not I restrictions. QuikChange II XL mutagenesis kit (Agilent) was used to make the 76K point mutation. Capsid fusion reporter alphaviruses expressing enhanced green fluorescent protein (eGFP) reporter proteins as a cleavable in-frame fusion between the capsid and E3 proteins were constructed essentially as described previously, except that the QuikChange II XL mutagenesis kit (Agilent) was used to insert a PCR fragment at the capsid/E3 junction that encoded the first five amino acids of E3 fused in frame to reporter genes followed by the TaV 2A-like protease, as described in Sun et al. [27]. All primers used for mutagenesis are listed in S4 Table. The VEEV TrD tripartite replicon system has been previously described [28,69] and modified by cloning of the eGFP gene under control of the authentic VEEV Trinidad Donkey (TrD) 26S subgenomic promoter. Replicon, capsid, and glycoprotein helper RNAs derived from VEEV TrD were electroporated into BHK cells and supernatants were harvested at 24 hours post electroporation followed by pelleting by centrifugation over a 20% sucrose in TNE cushion at 24,000rpm for 16–20 hours. Replicon single-round infectious units were determined by fluorescence microscopy of infected BHK cells. Deep Vent DNA polymerase (NEB) was used in all PCR reactions.

### Genome-to-PFU calculations

All viruses were titrated in triplicate on BHK-21 cells via standard plaque assay to determine PFU/mL. An aliquot from the same virus stock was used to determine genome numbers by adding 200uL of virus stock into 800uL Trizol Reagent for RNA extraction using 1-bromo-3-chloropropane (BCP) and isopropanol. Reverse transcription was then performed on the resulting RNA using an Invitrogen First-strand cDNA Synthesis M-MLV Reverse Transcriptase kit and a primer specific to the nonstructural protein 2 (nsP2) coding region of the appropriate virus. The 2X Fast TaqMan Universal PCR Master Mix, No AmpErase UNG (Applied Biosystems) kit was used alongside an appropriate nsP2 forward primer, reverse primer, and probe (S4 Table) to quantify genomic vRNA via qRT-PCR on a QuantStudio Flex-6 thermocycler (software version v1.7.2). A standard curve generated from in vitro transcribed virus RNA was used to determine the genome copies per mL of stocks. Genome to PFU ratios were calculated by dividing the average genome copy number/mL by PFU/mL. Higher ratios indicate reduced infectivity per virus particle on the specified cell type.

### Virus infectivity assays

For these assays, we used chimeric viruses (SINV/VEEV ZPC738) derived from cDNA clones that express Sindbis virus (SINV) nonstructural proteins and RNA replication control structures, and express VEEV structural proteins [70,71].

To test virus susceptibility to the absence of GAGs, eGFP-expressing chimeric viruses were serially diluted in virus diluent (PBS containing cations supplemented by 1% donor bovine serum (DBS), 100U/mL penicillin, and 0.5mg/mL streptomycin) then were used to infect control CHO-K1 cells, GAG-deficient CHO-pgsA-745 cells, or HS-deficient CHO-pgsD-677 cells. Cells were infected for 1hr at 37°C before an immunodiffusion-grade agarose overlay was applied, then cells were incubated for 18–20 hours before fluorescent plaques were visualized. Percent infectivity for each condition was calculated compared to the CHO-K1 controls for each virus.

To test whether mutant viruses are susceptible to increasing ionic conditions, viruses were serially diluted in RPMI-1640 buffer supplemented with increasing concentrations of NaCl to reach the listed concentrations, then CHO cells were infected with the diluted eGFP-expressing chimeras. Cells were infected as above except that fluorescent plaques were visualized after 2 days of incubation at 37°C. Percent infectivity for each virus was compared to RPMI-1640 only control, which has a salt concentration of around 102mM.

To test virus susceptibility to enzymatic digestion of GAGs on cells, BHK-21 cells were washed once with PBS containing cations, pre-treated with increasing concentrations of Heparinase II (0, 0.5, and 1 IU/mL, New England BioLabs) [72,73] for 1hr at 37°C, then washed twice with PBS containing cations before infection with eGFP-expressing chimeric virus serially diluted in virus diluent. Cells were infected as above and fluorescent plaques were visualized at 18–20hpi. Percent infectivity for each condition was calculated compared to the 0 IU/mL control group.

### Cellular binding assays

To determine ability of mutant viruses to bind to GAGs on cell surfaces, CHO-K1 and CHO-pgsA-745 cells were seeded in 24-well plates at 3x10^5^ cells/well the previous day. Cell monolayers were washed twice with PBS without cations while being chilled on ice, inoculated with equal genomes of chimeric eGFP reporter viruses, then placed on a nutator in a cold room for 90 minutes. After incubation, cells monolayers were washed 4 times with ice cold virus diluent to remove unbound virus. Trizol Reagent was then added to each well to lyse cell monolayers. RNA was extracted from samples using BCP and isopropanol, followed by reverse transcription and qRT-PCR as above. Primers targeting SINV nsP2 and the housekeeping gene *Mmadhc* (S4 Table) were used. Fold-change was directly quantified between nsP2 and *Mmadhc* levels, then percent binding compared to CHO-K1 was calculated for each virus.

### Growth curves

BHK-21 or RAW264.7 cells were infected in triplicate with WT and mutant viruses at an MOI 1. Cells were incubated at 37°C for 1hr, then washed with PBS with cations three times. Appropriate media was added to each well, then cells were incubated at 37°C for the duration of the experiment. Supernatants were sampled at each timepoint for infectious virus quantification via plaque assay on BHK-21 cells and/or genome copy quantification via qPCR. Aliquots were either stored at -80°C before plaque assay titration or added into Trizol Reagent and frozen at -80°C. Viral RNA was quantified with qRT-PCR as mentioned above, with primers targeting VEEV ZPC738 nsP2 containing a T7 promoter tag (S4 Table).

### Mouse infections

For all mouse infections with replication-competent viruses, groups of 5 mice were inoculated subcutaneously (s.c.) in both rear footpads with genomes equivalent to 1000 PFU of ZPC738 WT virus (determined by qRT-PCR). For mouse infections with non-propagative replicons, groups of 5 mice were infected s.c. in both rear footpads with genomes equivalent to 1x10^5^ infectious units (IU) of VREP (determined by qRT-PCR). Wild-type CD-1 mice were used for all experiments, except signaling pathway experiments in which knockout mice (MyD88-/-, TLR7-/-, MAVS-/-, TLR3-/-, IRF7-/-) and appropriate control mice (C57BL/6J for MyD88-/-, TLR7-/-, and IRF7-/- mice; C57BL/6x129 for MAVS-/- and TLR3-/- mice) were used.

### Mouse serum and tissue analysis

Serum samples were collected via submandibular vein at the indicated timepoints. Serum IFN-α levels were determined by the VeriKine™ Mouse IFN Alpha ELISA Kit, serum infectious virus titers were determined by plaque assay on BHK-21 cells, and proinflammatory cytokine and chemokine levels were determined by the Invitrogen ProcartaPlex Mouse Cytokine and Chemokine Convenience Panel 26-Plex kit.

For quantification of virus genome copies in lymph nodes, spleens, or footpads, mice were euthanized at the indicated timepoints, tissues were collected, then placed into Trizol Reagent. Samples were homogenized and virus in tissues were quantified by qRT-PCR as above with primer targeting VEEV ZPC738 nsP2 containing a T7 promoter tag (for virus infections) or targeting VEEV TrD nsP2 containing a T7 promoter tag (for replicon infections) (S4 Table).

### Lymph node imaging

At 8hpi, mice were euthanized and draining popliteal lymph nodes were collected and fixed in 4% paraformaldehyde for at least 24 hours. After fixation, tissues were dissected under a dissection microscope and placed into 70% ethanol for storage. Tissues were paraffin embedded, then sectioned. Hematoxylin and eosin were used to stain tissue sections to visualize histology. After blocking in 1% bovine serum albumin (BSA), serial sections were then stained with the following primary antibodies: anti-CD3e (ab16669, Abcam, SP7 clone, 1:150), BD Pharmingen biotin rat anti-B220 (Cat#553086, BD Biosciences, RA3-6B2 clone, 1:150), and Venezuelan equine encephalomyelitis virus immune ascitic fluid (Cat#VR-1249AF, ATCC, 1:300); and the following secondary antibodies: AlexaFluor 488 AffiniPure donkey anti-rabbit IgG (H + L) (Cat#711-545-152, Jackson Immunoresearch, 1:300), Rhodamine Red X (RRX) AffiniPure Fab Fragment Donkey Anti-Mouse IgG (H + L) (Cat#715-297-003, Jackson Immunoresearch, 1:100), and Strepavidin DyLight649 (Cat#SA-5649–1, Vector Laboratories, 1:750). SlowFade Gold with DAPI mounting media was used for counterstain. Images were acquired on a Nikon ECLIPSE Ti2-E confocal microscope. Images were processed and quantified using ImageJ (NIH) and Nikon Elements software.

### Flow cytometry

At 8hpi, mice were euthanized and draining popliteal lymph nodes were collected and placed into RPMI media. Immediately after collection, tissues were enzymatically dissociated with Liberase TL Research Grade (Cat#05401020001, Millipore Sigma) and Deoxyribonuclease I from bovine pancreas (Cat#: DN25–10MG, Millipore Sigma) and single cell suspensions were made by manually dissociating the tissue through a 70um filter. Cells were stained with BD Horizon Fixable Viability Stain 780 (BD Bioscience, Cat#: 565388, 1:1000), then fixed with 4% paraformaldehyde for 30 minutes. After fixation, mouse CD16/CD32 Fc block was added to the samples (clone 2.4G2 (ROU), BD, #553142, 1:200). Cells were stained overnight (16–20 hours) with the following antibodies: anti-CD45 BUV805 (clone 30-F11, BD, #568336, 1:4000), anti-CD3 BUV496 (clone 17A2, BD, #569671, 1:100), anti-CD4 RB705 (clone GK1.5, BD, #570258, 1:4000), anti-CD8a R718 (clone 53-6.7, BD, #566985, 1:20), anti-B220 BUV395 (clone RA3-6B2, BD, #563793, 1:200), anti-CD11c BV421 (clone N418, BD, 565451, 1:2000), anti-CD11b APC (clone M1/70, Tonbo Biosciences, #20–0112-U100, 1:8000), anti-CD317 RY775 (clone 927, BD, #770653, 1:2000), anti-Ly6C RB780 (clone AL-21, BD, #568739, 1:2000), and anti-F4/80 RY586 (clone T45-2342, BD, #753430, 1:2000). All antibodies were titrated to optimized concentrations for experimental conditions. Samples were run on a BD FACSymphony A5 SE spectral flow cytometer, then analyzed on FlowJo (V10.10.0). Gates were created on fluorescent minus one (FMO) controls for each antibody and applied equally to all samples.

### CD317 depletion

Anti-CD317 (anti-BST2, clone 120G8.04, Novus Bio, #DDX0390-HD10) and purified rat IgG1 (clone M1/70, bioXcell, #BE0007) antibodies were used to deplete plasmacytoid dendritic cells or used as an isotype control, respectively. 4-week-old CD-1 mice were treated intraperitoneally (i.p.) with 100ug/mouse of corresponding antibody both 48 and 24 hours prior to infection. On challenge day, 5 mice per treatment group were infected s.c. with 1000 PFU of ZPC738 WT, 3 mice per treatment group were mock infected with 10uL of OptiMEM, and 3 mice were sacrificed. Spleens and popliteal lymph nodes were taken from euthanized mice, made into single cell suspensions, then stained and analyzed via flow cytometry to confirm depletion of target cell types, as described above.

### Statistical analysis

GraphPad Prism version 10.3 was used to determine statistical significance for all experiments. Statistical test used for each figure is denoted in figure legends. *In vitro* experiments were conducted in technical triplicate and repeated at least twice with similar results. For mouse experiments, each group contained n = 5 mice unless stated otherwise, and studies were performed at least twice with similar results. The specific test for each dataset is described in respective figure legends and was selected by the number of comparison groups and variance of the data. All error bars represent mean ± SD. Alpha values of 0.05 were used to determine significance. *(p < 0.05), **(p < 0.01), ***(p < 0.001), ****(p < 0.0001)

### Visium HD assay, data processing, and segmentation

After infection with 1000 PFU of ZPC738 eGFP TaV or mock infection with equal volume of OptiMEM, mice were euthanized and draining popliteal lymph nodes were collected at 8 or 16hpi. Tissues were fixed in 4% PFA for 24 hours, then placed into 70% ethanol for storage. Tissues were then embedded in paraffin. Samples were assessed for RNA quality, and all FFPE samples had a DV200 score above the minimum threshold ≥30%. 4–8 lymph nodes per group (mock or infected) were included in the assay. Spatial transcriptomic libraries were generated with Visium HD Mouse Transcriptome gene expression kit (10x Genomics: 1000676). 5-micron FFPE sections were placed on Superfrost Plus slides (Fisherbrand, #22-037-246) and processed through the 10x Visium HD for FFPE protocol according to the manufacturer’s instructions (CG000685). H&E images were taken at 40x magnification using a Leica Aperio CS2 slide scanner (Leica Biosystems). A Spike-In Pool Working Stock was made for the custom probes (24nM/probe) and 10uL were added to the Probe Hybridization Mix. Library QC was completed with an Agilent TapeStation 4150. Libraries were normalized and pooled to 2nM prior to loading on an Illumina NextSeq 2000, using a P4 100 flow cell with a target of a minimum of 275 million reads per sample for transcriptomic libraries. Visium HD reads were aligned to mouse transcriptome. The mouse mm10 (GRCm38) and VEEV (ZPC738-eGFP-Tav) combination genome was generated by merging the fasta files and.gtf files from each genome and using STAR [74] to generate a reference combo genome for alignment with spaceranger 3.0.1. The VEEV designed probes were added to the 10x Visium HD mouse probe set v2 list for transcript identification during alignment. Binning was performed using Space Ranger’s cell segmentation algorithm for all downstream analyses to balance spatial resolution with transcriptomic depth. Output filtered feature-barcode matrices and spatial coordinates were used for all subsequent analyses.

### Data loading, quality control, and normalization

Processed Space Ranger outputs were imported into R using the Seurat [75–77] package (v5.3.1.9999). For each sample, spots were filtered to retain those with a minimum of 50 unique molecular identifier (UMI) counts to exclude low-quality or dying cells. Lymph node regions of interest were manually annotated and isolated from full tissue sections based on H&E morphology and spatial clustering, and only spots falling within lymph node boundaries were retained for downstream analysis. Gene expression counts were normalized using NormalizeData with default parameters, regressing out mitochondrial content percentage, which simultaneously performs variance stabilization and feature selection.

### Dimensionality reduction, clustering, and integration

Principal component analysis was performed on the top 2,000 variable genes identified by FindVariableFeatures. The top 10 principal components were used as input for Harmony batch correction to integrate across all samples and time points, correcting for sample-level technical variation while preserving biological signal. The Harmony [78]-corrected embeddings were used to construct a shared nearest-neighbor graph using FindNeighbors, followed by unsupervised Leiden clustering via FindClusters. Final clustering resolution was selected based on cluster stability and correspondence with known lymph node compartment architecture as validated by marker gene expression and H&E morphology. UMAP dimensionality reduction was computed on Harmony embeddings for visualization purposes.

### Cell type deconvolution

Cell type composition of each segmented bin was estimated using Robust Cell Type Decomposition [35] (RCTD) implemented in the spacexr R package. A single-cell RNA sequencing reference atlas of murine lymph node immune cell populations was used as the deconvolution reference [36,79], comprising of annotated populations including B cells, T cells, NK cells, plasmacytoid dendritic cells, conventional dendritic cells (cDC1, cDC2), monocytes, macrophages, neutrophils, and stromal subsets. This atlas was created by separately administering 86 cytokines into mice, then performing scRNA-seq on the DLNs. RCTD was run in doublet mode, which is optimized for high-resolution platforms such as Visium HD where individual bins frequently capture signal from more than one cell. For spatial visualization, each bin was assigned to the cell type with the highest predicted proportion. For downstream analyses stratified by cell type and infection status, only bins classified as singlets by RCTD were retained, ensuring high-confidence single-cell-type assignments.

### Differential gene expression analysis

Pseudobulk differential expression analysis was performed to compare Infected versus Mock conditions at each time point independently (8 hpi and 16 hpi). Spots from all lymph nodes per condition were aggregated by sample to generate pseudobulk counts, and DESeq2 [80] was used to model count data with a negative binomial distribution. The design formula included infection status as the primary variable of interest. Genes were considered significantly differentially expressed at an adjusted p-value threshold of < 0.05 (Benjamini-Hochberg correction) and an absolute log2 fold change > 1. Results were visualized as volcano plots using ggplot2, with significant genes highlighted and key cytokine and interferon genes labeled.

### Pathway enrichment analysis

Gene set enrichment analysis [81] (GSEA) was performed on pre-ranked gene lists using gene sets from the Gene Ontology categories Biological Process (BP), Cellular Component (CC), and Molecular Function (MF). Normalized Enrichment Scores (NES) and Benjamini-Hochberg-adjusted p-values were computed for each pathway. The top upregulated and downregulated pathways ranked by NES were visualized as horizontal bar plots for each time point comparison, with bars colored by NES direction.

### Cytokine and interferon expression analysis

A manually curated gene list of cytokines, chemokines, and interferon-stimulated genes was assembled, encompassing type I interferons (*Ifna* family, *Ifnb1*), type III interferons (*Ifnl2, Ifnl3*), and key inflammatory mediators (*Cxcl10, Ccl2, Ccl7*, *Tnf, Il6,* and others). For dot plot visualization of cytokine expression by cell type, only cells in the Infected samples (Infected_8h and infected16h) were stratified by RCTD-assigned cell type and further subdivided by infection timepoint. Within each cell type group, the mean scaled expression and percentage of spots with detectable expression (count > 0) were calculated for each gene. This was done separately for only actively VEEV-replicating cells, only non-VEEV-replicating cells, and both actively and non-VEEV-replicating cells. Dot plots were generated using the DotPlot function in Seurat or custom ggplot2 [82] implementations, with dot color representing average scaled expression and dot size representing percent expression. For spatial visualization of type I IFN and general cytokine/chemokine, per-spot module scores were calculated using AddModuleScore in Seurat for the respective gene lists, and scores were plotted onto tissue coordinates using SpatialFeaturePlot with consistent color scales across samples.

### Viral cell classification and actively versus Non-VEEV-replicating cell analyses

To identify virally infected spots, UMI counts for VEEV structural gene E1 and non-structural gene nsP4 were extracted from the Space Ranger output using custom viral genome reference. UMIs are molecular barcodes that allow quantification of individual transcripts in sequencing libraries; thus, an assumption can be made that 1 UMI = 1 transcript copy. As described previously using qRT-PCR, subgenomic-to-genomic ratios of virus gene expression during alphavirus infection have been used to measure active replication in various sample types [37–40]. E1 and nsP4 are the final genes in their respective open reading frames; therefore, using these subgenomic/genomic parameters ensures that transcripts detected are more likely to be full genome transcripts, not genome fragments. The UMI(E1), UMI(nsP4), and E1:nsP4 values were empirically determined to minimize false positives from ambient RNA or low-level probe cross-reactivity, while still retaining bona fide infected cells as validated by correspondence with FCA of known infection-negative cells and IHC of VEEV-infected PLNs. Of note, increasing nsP4 and E1:nsP4 values had minimal impact on the estimated infection rates; thus, the lowest values (UMI(nsP4) ≥ 1 and E1:nsP4 > 1) were chosen as conservative filters to ensure detection of replicating virus. UMI(E1) values were adjusted until they identified ~2% of B cells and ~1% of T cells as infected (as determined by FCA) and ~10% the total PLN area as VEEV-positive (as determined by IHC), which occurred around UMI(E1) ≥ 50. Thus, spots were classified as infected if they met all three criteria: E1:nsP4 > 1, UMI(E1) ≥ 50, and UMI(nsP4) > 1.

The percentage of infected spots among total spots for all lymph nodes in Infected samples were calculated and displayed as pie charts. For analysis of gene expression between actively VEEV-replicating and non-VEEV-replicating cells, infected spots were overlaid on spatial plots of individual gene expression. Actively VEEV-replicating spots were colored yellow, non-VEEV-replicating spots white, and expression of each gene of interest mapped onto a blue sliding scale. Spots meeting both the infection criteria and expressing the gene of interest were shown in green. Stacked bar graphs adjacent to each spatial plot were computed by summing total counts of the gene of interest across all spots, partitioned into actively and non-VEEV-replicating cell contributions, with exact percentages labeled and mean scaled expression for each group reported beneath each bar. The proportion of cytokine transcripts originating from actively VEEV-replicating cells and non-VEEV-replicating cells were displayed as pie charts for 8hpi and 16hpi timepoints.

### Visualization and statistical reporting

All spatial plots were generated using SpatialFeaturePlot and SpatialDimPlot from Seurat, with consistent color palettes and spot size parameters across time points to enable direct visual comparison. Representative lymph nodes were selected for spatial figure panels based on median total UMI count and median cluster composition closest to the sample-level mean, ensuring representativeness of the full dataset. Analyses requiring full dataset statistics (differential expression, GSEA, dot plots) incorporated all lymph nodes from all biological replicates. All plots were customized in R. Statistical comparisons for differential expression used DESeq2’s Wald test with Benjamini-Hochberg multiple testing correction as described above. All analyses were performed in R version 4.3.

## Supporting information

S1 Fig Heparan sulfate binding ability and miR142-3p-dependent replication restriction can be conferred to VEEV *in vitro.* (A) Growth curve of WT and 76K viruses in BHK-21 cells (n = 6, 2 independent experiments). (B) Relative infectivity on CHO-K1, GAG-deficient CHOpgsA-745, and HS-deficient CHOpgsD-677 cells (n = 15 (CHO-K1) or n = 9 (CHO-pgsA/pgsD); 5 (CHO-K1) or 3 (CHO-pgsA/pgsD) independent experiments). (C) Relative infectivity on CHO-K1 cells incubated in media with increasing NaCl concentrations (n = 9, 3 independent experiments). (D) Relative infectivity on BHK-21 cells treated with increasing concentrations of heparinase-II (n = 9, 3 independent experiments). (E) GE-to-PFU ratios calculated for ZPC738 WT and 76K. Each datapoint represents 2 averaged values from an independent experiment (n = 3, 3 independent experiments). (F) Relative binding on CHOK1 and GAG-deficient CHOpgsA-745 cells measured via qRT-PCR (n = 9, 3 independent experiments). (G) Growth curves in BHK-21 and RAW264.7 cells (n = 6, 2 independent experiments). Statistical significance determined via 2-way ANOVA with Sidak’s (A), Tukey’s (B, D, F), or Dunnett’s (C, G) multiple comparison test, or unpaired t-test (E).
(TIF)

S2 Fig A heparan sulfate binding mutation and miR142-3p binding sites restrict VEEV replication in the draining lymph node. (A) Spleens collected at 8, 16, and 24hpi from infected CD-1 mice, then analyzed for viral load via qRT-PCR (n = 10, 2 independent experiments). (B) Footpad tissues collected at 8hpi, then analyzed for viral load via qRT-PCR (n = 5, 1 independent experiment). (C) CD-1 mice were infected subcutaneously split between footpads with VREP, then PLNs and spleens were collected at 8hpi and analyzed for viral load via qRT-PCR (n = 10, 2 independent experiments). (D-E) Capsid-to-nsP2 ratios were calculated via qRT-PCR for 8 and 16hpi (D) PLN and (E) spleen data collected in Fig 1E-1F (n = 10, 2 independent experiments). (F) 8hpi PLNs were stained with anti-VEEV antibodies at both 10X (top, scale bar = 200um) and 60X (bottom, scale bar = 5um) magnification (n = 4–6, 1 independent experiment). (G) 8hpi PLNs were stained with DAPI, anti-B220, anti-CD3, and anti-VEEV antibodies (n = 4–6, 1 independent experiment, 60X magnification). **(H)** Area of VEEV staining. Each dot represents a random field of view taken at 60X magnification around the subcapsular region of the PLN (n = 10 between 4 lymph nodes, 1 independent experiment). **(I)** Percent of total staining area that colocalizes with VEEV staining in WT samples for both B220+ and CD3 + cells. Quantified from 60X images. Each dot represents a random field of view taken around the subcapsular region of the lymph node (n = 10 split between 4 lymph nodes). **(J)** CD-1 mice were infected subcutaneously in the footpad with equal genomes of ZPC738 WT and VREP, serum was collected at 8hpi, and IFN-alpha was measured via ELISA. n = 10 mice from at least 2 independent experiments. Statistical significance determined via 2-way ANOVA with Tukey’s multiple comparison test **(A)**; unpaired t-test **(C, J)**; Welch’s t-test **(D, E)**; or one-way ANOVA with Tukey’s **(B)** or Sidak’s **(H, I)** multiple comparison test.
(TIF)

S3 Fig CD317 depletion results in reduced proinflammatory cytokine responses at 24hpi. (A) Flow cytometry validation of CD317 depletion in the lymph node and spleen from mice treated with anti-CD317 antibody. Values are normalized to the mean percentage of CD317 + cells in isotype control samples (n = 8, 3 independent experiments). (B) Bar graphs showing select cytokines and chemokines at 8hpi (left) and 24hpi (right) (n = 5, 1 independent experiment). All error bars represent SD. Significance was determined via one-way ANOVA (A; for B, within cytokine groups) with Tukey’s multiple comparison test.
(TIF)

S4 Fig Spatial plots show rapid spatial reorganization and proinflammatory upregulation in the lymph node. Spatial plots of (A) H&E-stained tissues, (B) unsupervised clustering, which groups spots with transcriptionally similar profiles, (C) general cytokine and chemokine expression, and (D) type III IFN expression in a representative lymph node from each sample. White arrows in (A) denote areas of decreased cellular density and potential cell death. Scales for (C, D) represent log-normalized expression values.
(TIF)

S5 Fig Spatial transcriptomics accurately identifies infected cell identities within the lymph node. (A) Spatial plots showing quantification of each viral gene included in the custom VEEV probe set on a representative 8hpi infected lymph node. Scales represent log-normalized expression values. (B) Bar graph showing number of “infected” cells when classified with E1:nsP4 > 1, > 5, > 10, and >50. (C) Spatial plot showing number of “infected” cells (red) when classified with E1:nsP4 > 1, > 5, and >10. (D) Spatial plots showing infected cells (red) classified by UMI(E1) and UMI(nsP4) levels. (E-G) Flow cytometry data showing (E) percent of each cell type that is infected, (F) pie chart of total cell composition, and (G) gating strategy for cell identification. Gating strategy is described using a representative mock sample, with mock and infected samples shown for each infection-determining gate (n = 10, 2 independent experiments). (H) 8hpi PLNs were stained with anti-VEEV antibodies to visualize virus infection within the PLN at 10X magnification. Area of VEEV staining was quantified using Nikon Elements Analysis software. Each datapoint represents an individual PLN (n = 4, 1 independent experiment).
(TIF)

S6 Fig Active VEEV infection in the subcapsular LN region decreases from 8hpi to 16hpi. (A) Comparison between ZPC738 WT genome copies in the PLN at 8, 16, and 24hpi. Data originally shown in Fig 1E and reanalyzed for this figure. Significance was determined via one-way ANOVA with Tukey’s multiple comparison test. (B) Spatial plot showing nsP4 expression color-coded by expression level. Scale represents log-normalized expression values. (C) Bar graph showing average gene expression for select genes at varying nsP4 thresholds at 8hpi (top) and 16hpi (bottom). Zero cells were identified at 16hpi with nsP4 ≥ 50 and are thus annotated with “no available data”.
(TIF)

S1 Table Proinflammatory cytokines and chemokines induced after mutant VEEV infection. Values are indicated in pg/mL. Range of replicate values for each group are included in parentheses. Significant differences are indicated with asterisks, compared only to appropriate controls (WT/76K; miR + /miR + MM), and were calculated using one-way ANOVA with Tukey’s multiple comparison test within cytokine groups. Mock datapoints included were originally collected and published in Trobaugh et al., 2019 [9].
(TIF)

S2 Table Proinflammatory cytokines and chemokines induced 24 hours after infection of genetic knockout mice with VEEV. Values are indicated in pg/mL. Range of replicate values for each group are included in parentheses. Significant differences are indicated with asterisks, compared only to appropriate controls (B6/MyD88-/-; B6/129/MAVS-/-), and were calculated using one-way ANOVA with Tukey’s multiple comparison test within cytokine groups.
(TIF)

S3 Table Proinflammatory cytokines and chemokines induced after infection of mice treated with anti-CD317 antibody. Values are indicated in pg/mL. Range of replicate values for each group are included in parentheses. Significant differences are indicated with asterisks, compared only to isotype controls at each timepoint, and were calculated using one-way ANOVA with Tukey’s multiple comparison test within cytokine groups.
(TIF)

S4 Table Primers and probes used in this study. Primer and probe sequences are listed from 5’-3’. Assays utilizing each primer and probe are listed beside each entry.
(TIF)
